# Defective Thyroglobulin: Cell Biology of Disease

**DOI:** 10.3390/ijms232113605

**Published:** 2022-11-06

**Authors:** Xiaohan Zhang, Crystal Young, Yoshiaki Morishita, Kookjoo Kim, Omer O. Kabil, Oliver B. Clarke, Bruno Di Jeso, Peter Arvan

**Affiliations:** 1 Division of Metabolism, Endocrinology & Diabetes, University of Michigan, Ann Arbor, MI 48105, USA; 2 Department of Molecular & Integrative Physiology, University of Michigan, Ann Arbor, MI 48105, USA; 3 Division of Diabetes, Department of Internal Medicine, Aichi Medical University, Nagakute 480-1195, Japan; 4 Department of Physiology and Cellular Biophysics, Columbia University Irving Medical Center, New York, NY 10032, USA; 5 Department of Natural Sciences, Lindenwood University, Saint Charles, MO 63301, USA; 6 Department of Biological and Environmental Sciences and Technologies, University of Salento, 73100 Lecce, Italy

**Keywords:** endoplasmic reticulum, ER stress, secretory pathway, hormonogenesis, cell death

## Abstract

The primary functional units of the thyroid gland are follicles of various sizes comprised of a monolayer of epithelial cells (thyrocytes) surrounding an apical extracellular cavity known as the follicle lumen. In the normal thyroid gland, the follicle lumen is filled with secreted protein (referred to as colloid), comprised nearly exclusively of thyroglobulin with a half-life ranging from days to weeks. At the cellular boundary of the follicle lumen, secreted thyroglobulin becomes iodinated, resulting from the coordinated activities of enzymes localized to the thyrocyte apical plasma membrane. Thyroglobulin appearance in evolution is essentially synchronous with the appearance of the follicular architecture of the vertebrate thyroid gland. Thyroglobulin is the most highly expressed thyroid gene and represents the most abundantly expressed thyroid protein. Wildtype thyroglobulin protein is a large and complex glycoprotein that folds in the endoplasmic reticulum, leading to homodimerization and export via the classical secretory pathway to the follicle lumen. However, of the hundreds of human thyroglobulin genetic variants, most exhibit increased susceptibility to misfolding with defective export from the endoplasmic reticulum, triggering hypothyroidism as well as thyroidal endoplasmic reticulum stress. The human disease of hypothyroidism with defective thyroglobulin (either homozygous, or compound heterozygous) can be experimentally modeled in thyrocyte cell culture, or in whole animals, such as mice that are readily amenable to genetic manipulation. From a combination of approaches, it can be demonstrated that in the setting of thyroglobulin misfolding, thyrocytes under chronic continuous ER stress exhibit increased susceptibility to cell death, with interesting cell biological and pathophysiological consequences.

## 1. Role of the Endoplasmic Reticulum in Secretory Protein Synthesis and Intracellular Transport

The protein biosynthetic apparatus of cells is tightly linked to the differentiated phenotype of eukaryotic cells and tissues, by regulating the abundance and delivery of new proteins to the various organelles that control cellular function. Successful protein targeting to specific intracellular and extracellular destinations is dependent upon targeting information encoded with the polypeptide structure, and cellular machinery designed to ‘read’ that targeting information. The failure to either properly present or read such information provides an important entrance into the pathobiology of disease [1].

The endoplasmic reticulum (ER) is one of the largest organelles in eukaryotic cells. ER function contributes to multiple activities including protein synthesis, folding, and transport, as well as lipid and steroid synthesis, carbohydrate metabolism, and calcium storage [2], whereas ER dysfunction contributes to a large variety of diseases [3]. Proteins that follow the classical protein secretion pathway begin their life within the ER, and they remain intracellularly enclosed within specific membrane-bound compartments and vesicular-tubular transport intermediates as they migrate through the cytoplasm; at each successive station along this route, iterative decisions are made on the basis of anterograde transport signal-mechanisms (e.g., [4]) and retention signal-mechanisms (e.g., [5]). The process begins with translation of secretory proteins on the cytosolic side of the ER membrane; ribosomes reading secretory protein mRNAs are recruited to the ER membrane via recognition and binding of the of the signal peptide to the signal recognition particle (SRP) for delivery of the nascent polypeptide to the SRP receptor on the ER membrane [6]. The continuation of translation at the ER is coupled to co-translational translocation of the nascent polypeptide across the ER membrane [7]. During the translocation process, signal peptidase excises the short signal peptide [8], permitting subsequent steps in the nascent secretory maturation process within the ER lumen, including N-linked glycosylation [9], disulfide bond formation [10], and oligomerization [11]—each event stimulated via a number of ER molecular chaperones and folding enzymes [12,13]. These ER resident proteins contribute to a quality control function such that properly folded secretory proteins are allowed anterograde advance towards the Golgi complex, but those proteins that do not achieve an acceptable conformation are retained within the ER [14,15] and can subsequently become available for ER-associated degradation (ERAD, [16]), degradative ER-phagy [17], or may remain undegraded [18].

These general principles are not unique to thyrocytes, yet these cells do exhibit a uniquely high expression of a subset of gene products that encode thyroid-specific function, leading to the synthesis of thyroid hormones. Although thyroid hormone synthesis in lower organisms takes place in the absence of an organized thyroid gland architecture, in vertebrates, thyroid hormone synthesis occurs in thyroid follicles, each representing the basic functional unit of the thyroid gland, and each expressing the thyroglobulin (Tg) protein [19,20]. The thyroid follicle is composed of an epithelial monolayer of polarized thyrocytes with the basolateral surface facing the bloodstream and the apical surface delimiting a central cavity known as the follicle lumen; and it is primarily in this apical extracellular space wherein protein iodination leading to the formation of mono-iodotyrosine (MIT), di-iodotyrosine (DIT), and the thyroid hormones thyroxine (T_4_) and tri-iodothyronine (T_3_) actually takes place [21,22]. In the vertebrate thyroid gland, efficient production of thyroid hormones requires the proper synthesis, folding, and transport of Tg, the most abundantly expressed thyroid-specific gene product. Differentiated thyrocytes are powerfully dedicated to the biosynthesis of the Tg glycoprotein [23], which normally accounts for more than half of all the protein in the thyroid gland [24,25]. The newly-synthesized Tg protein is delivered via a short signal peptide to the ER lumen [26], wherein the protein acquires multiple N-linked glycans [27] and disulfide bonds [28] en route to formation of folded Tg monomers [19]. Due to the large size of the Tg polypeptide (~2750 residues), the post-translational Tg folding process is slow [29], involving a series of Tg folding intermediates [23], accompanied by extensive interaction with ER resident proteins participating in protein folding and quality control [30]. Perhaps unsurprisingly, amongst the most important of these ER resident proteins are BiP (the ER member of the hsp70 family [29]) as well as ER oxidoreductases ERp57, PDI, P5, and ERp72 [31,32], which are all highly abundantly expressed in the ER lumen. Most likely, different chaperone complexes and subcomplexes are involved with distinct regions of the Tg protein, and kinetically at different times during the Tg folding process [33]. Ultimately, significant Tg monomer folding is required for proper presentation of the Tg dimer interface necessary for homodimerization [34,35], which is a step that occurs largely, if not exclusively, prior to anterograde export from the ER [36].

## 2. Tg Folding and TG Mutations That Trigger Misfolding

The organization of the *TG* gene and its 48 exons [37], as well as proven or predicted mutations that affect the Tg protein sequence [38] have been well described elsewhere. Here, we offer a few additional observations that have emerged from recent studies.

**Figure 1:**
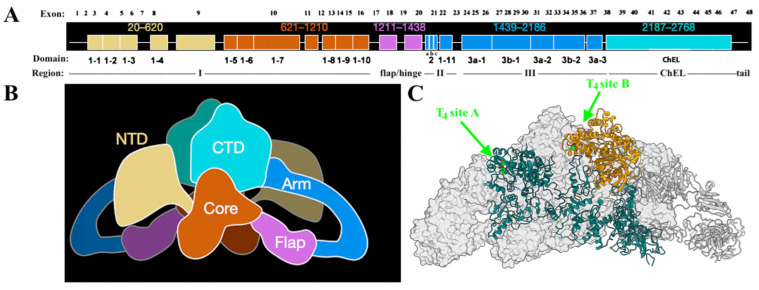
Thyroglobulin Organization (panel (**A**), exons listed above), Primary Structure (Panel (**A**), regions and domains shown below); Tertiary Structure from cryo-EM model (panel (**B**)), and the two most-utilized T_4_ formation sites (panel (**C**), in bright green).

After cleavage of its signal peptide, Tg is initially synthesized as a monomeric protein (whose primary structure has been divided into regions) although the final native structure is actually a homodimer [36]. The first ~80% of the Tg protein has been described informally as I-II-III [39] because it contains the first 10 Tg type 1 repeats in region I (each repeat is defined largely by the arrangement of cysteine residues that form internal disulfide bonds), separated by a flap/hinge region, from three short type 2 repeats, plus a final type 1 repeat in region II and followed by five Tg type 3 repeats in region III (Figure 1A; each of these repeats also forms internal disulfide bonds). The Tg protein concludes with the Cholinesterase-Like domain (ChEL, bearing three internal disulfide bonds) plus a short unique tail sequence (Figure 1A). Four recent papers have provided new insight into the three-dimensional structure of Tg (two human; two bovine) by modeling from cryogenic electron microscopy [28,34,40,41], and schematic of the 3D structure is shown in Figure 1B. Specifically, when the color-coded image of Figure 1B is considered alongside the color-coded primary structure of Tg in Figure 1A, it becomes apparent that globally, the monomer structure has an overall J-like shape, in which region I is roughly equally divided into an ‘N-terminal domain’ encoded by the first 9 exons (of a total of 48), and what has been called the ‘core domain’ is encoded by the next 7 exons. The following flap/hinge region essentially bisects the entire Tg monomer sequence, leading to an ‘arm domain’ encoded by 17 exons, which contains all of the Tg type 2 and type 3 repeats (as well as a single, 11th, type 1 repeat). The ‘C-terminal domain’ plus the short unique tail sequence together (encoded by the last 10 exons) contains one of the two preferred sites for the de novo formation of T_4_ as well as the one preferred site for the de novo formation of T_3_ [42]. Thus far, only one cryo-EM study has reported the successful simultaneous visualization of both of the two most-utilized sites for the de novo formation of thyroxine, including the most-important “site A” in the N-terminal domain, and the second-most- important “site B” in the ChEL domain (Figure 1C, [40]). The exons encoding these regionally distinct domains of Tg are likely to have distinct evolutionary ancestry, although the origins of these “pieces” of the Tg gene residing in invertebrates have not all been definitively identified [20].

What is known is that the expression of region I-II-III alone (product of the first 37 exons of Tg that linearly encompasses from the N-terminal domain through to the arm domain) yields a protein that is not competent for anterograde transport from the ER. Remarkably, co-expression of the C-terminal ChEL domain as an entirely independent secretory protein results in a tight association of region I-II-III with this separately-expressed domain, rescuing the anterograde transport of I-II-III, with the two components remaining associated after secretion from cells [39]. Moreover, the C-terminal ChEL domain is not only necessary but also sufficient for tail-to-tail dimerization as demonstrated both by sucrose gradient centrifugation and by the formation of an artificially constructed intermolecular disulfide bond that is spontaneously generated when a single unpaired cysteine is introduced into the short unique tail sequence—and this same cysteine can also engage in the same unique disulfide crosslink within the homodimer when it is similarly introduced into the sequence of full-length Tg [43]. Considerable monomer folding is needed for homodimerization (ordinarily noncovalent) in the thyrocyte ER, and this seems to be a precondition for passing the ER quality control requirements for Tg export [19]. With this in mind, it is not surprising that mutations affecting the Tg coding sequence in such a way as to impede the formation of disulfide bonds within the many repeating units—or the three-dimensional packing of the overall globular protein (Figure 1B)—result in a shared, common phenotype of defective export from the ER, with diminished delivery to the thyroid follicle lumen and, consequently, diminished iodination and thyroid hormonogenesis.

We note that one group has estimated the genetic frequency in the general human population of presumed likely pathogenic variants of *TG* (heterozygosity) at 1:320 people [44], and a second group has similarly estimated this frequency at 1:217 people [45]. Either way, the occurrence of individuals expressing misfolded Tg protein from at least one allele is likely to be rather common. However, hypothyroidism from defective thyroglobulin is inherited as an autosomal recessive trait [46]. These data provide genetic evidence that wildtype Tg expressed from a single allele, along with the natural plasticity and endocrine feedback built into the hypothalamic-pituitary-thyroid axis, is sufficient to provide for the body’s needs for thyroid hormone.

## 3. Tg Misfolding and Its Consequences in Cell Culture Models

Tg misfolding can be studied either in thyrocyte cell lines, or by use of a suitable vector for expression in non-thyroidal cells. The first approach requires perturbation of the folding of endogenous wildtype Tg, and the second approach employs transfection of heterologous cells. To achieve success with the first approach, interruption of Tg folding in thyrocytes can be achieved pharmacologically, whereas the second approach allows for mechanistic insight into the effect of specific mutations (that are known to be linked to congenital hypothyroidism in humans or animal models) on Tg protein folding and trafficking. Unsurprisingly, transient expression of mutant *TG* genes in such heterologous cells results in the misfolded Tg protein being trapped in the ER, unable to advance to the Golgi complex (and thus unable to be secreted) because of ER quality control [47]. Surprisingly, however, some mutant Tg may interact with and be partially rescued by co-expression with wildtype (i.e., in 293T cells), presumably via weak cross-dimerization between the more folding-competent wildtype Tg monomer and the less folding-competent mutant Tg [43,48]. 

Protein misfolding in the ER is continuously monitored through the activities of ER stress sensors IRE1, PERK, and ATF6, which transmit responses leading to repression of global translation while increasing the expression of ER chaperones (e.g., BiP) and protein folding factors, accompanied by increased ER-associated and autophagic degradation of misfolded proteins [49]. However, under conditions of unremitted ER stress [50], cell death pathways can be activated [51]. A CRISPR/Cas9-mediated disruption of the *TG* gene in PCCL3 cell clones leading to undetectable Tg synthesis results in a more than 30% decrease in basal levels of BiP mRNA, and reduced ER stress response or cell death in the face of tunicamycin [52], an inducer of ER stress that works by inhibiting all N-linked-glycosylation (including that of Tg, which is normally the major N-glycosylated protein of thyrocytes). These findings strongly suggest that the misfolding of Tg itself can be a major contributor to ER stress, ER stress response, and ER stress-related cell death in thyrocytes.

ER-mediated diseases including congenital hypothyroidism typically take months or years to become clinically apparent [11,53], which means that patients and animal models with mutant Tg suffer from chronic continuous ER stress in their thyrocytes. This is not an easy phenotype to model in cell culture, but it is possible to challenge PCCL3 thyrocytes pharmacologically with chronic tunicamycin treatment in order to mimic the chronic ER stress condition. Initially, adaptation to chronic tunicamycin exposure occurs by a suppressed expression of the tunicamycin transporter (encoded by Mfsd2a [54]), but this can be surmounted either by slow, stepwise increments of the tunicamycin dose over a series of cell culture passages—or by simply using CRISPR-mediated genetic deletion of Mfsd2a and then immediately and permanently challenging PCCL3 thyrocytes with the highest possible dose of tunicamycin. In either case, a thyrocyte culture model can be obtained that adapts and survives long-term in the presence of chronic, continuous ER stress [52]. The growth rate of such adapted thyrocytes appears relatively normal, and this may be consistent with the ability to grow a thyroid goiter in many humans and animal models expressing misfolded mutant Tg.

A proteomic analysis of cultured thyrocytes adapted to chronic continuous ER stress revealed many up-regulated proteins, indicating stimulation of ER chaperones, oxidoreductases, stress responses, and lipid biosynthesis pathways [52], some of which had already been observed in in vivo models [55]. Furthermore, ER stress-adapted PCCL3 thyrocytes exhibited up-regulated AMP-Kinase activity (suggested by increased phosphorylation of AMPK), and decreased mTOR activity (suggested by diminished phosphorylation of S6-kinase and protein translation)—and a chemical AMPK activator was found to decrease the death of acutely ER-stress-challenged PCCL3 cells—all appearing consistent with the activation of conserved cell survival/adaptation pathways [52]. Additionally, subtle signs of de-differentiation including diminished PAX8, FOXE1, and TPO protein levels, along with decreased thyroglobulin mRNA levels were also observed in chronically ER stress-adapted cultured thyrocytes [52]. Furthermore, among the adaptive responses of chronically ER-stressed, partially de-differentiated PCCL3 cells included an apparent suppression of the mRNA level of Cidea (cell death-inducing DFFA-like effector-A, a protein of lipid droplets), as well as a blunted Cidea mRNA response (and blunted cell death response) to a new and unrelated acute ER stress challenge. In contrast, an acute ER stress challenge of either PCCL3 cells or several non-thyroid cell lines results in an acute increase of Cidea mRNA levels, indicating that CIDE-A is a novel noncanonical ER stress marker, which may have implications for cell death (when it is elevated) and cell survival (when it is suppressed) [56]. As CIDE-A may downregulate AMPK activity [57,58] to promote stress-mediated autophagy and cell survival, these observations might help to explain thyrocyte survival in the face of chronic continuous ER stress derived from misfolded Tg protein [52]. 

## 4. Tg Misfolding and Its Consequences for Animal Models and Humans

In congenital hypothyroidism with biallelic *TG* mutation, negative feedback from the intact hypothalamic-pituitary-thyroid axis results in chronic and dramatic elevation of circulating TSH. A prolonged stimulation of thyrocyte proliferation [59] induces thyroid growth (goiter) both in animals [60,61] and in patients [46,62,63,64]. However, linkage analysis suggests that the *TG* gene is also linked to congenital hypothyroidism in some families without goitrogenesis [65].

**Figure 2:**
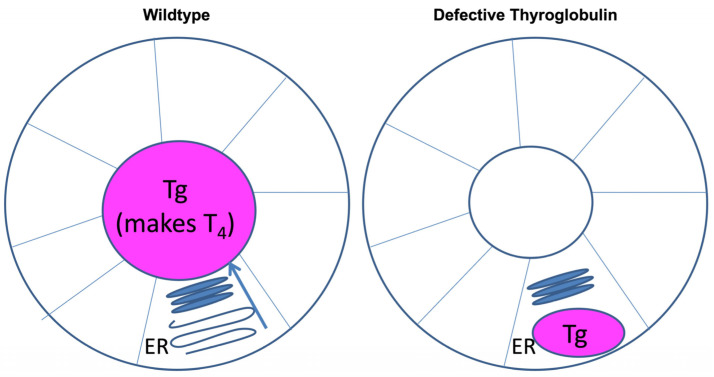
The distribution of thyroglobulin protein in thyroid follicles under conditions that are either wildtype (*left*), or in case of Hypothyroidism with Defective Thyroglobulin (*right*). In wildtype thyroid (*left*), Tg is synthesized in the ER of within the thyroid epithelial monolayer. From there, Tg undergoes intracellular trafficking leading to its secretion into the follicular lumen. Secreted Tg serves as the primary substrate of thyroid hormone biosynthesis, utilizing thyroidal iodination machinery that is located at the apical membrane. In hypothyroidism with defective Tg (*right*), misfolded Tg is blocked in intracellular transport from the ER, inducing ER stress with massive ER expansion. Tg remaining within the ER of the thyroid epithelial monolayer is unavailable for thyroid hormone biosynthesis.

Biallelic *TG* mutations, with or without goiter, have also been described in rodent models. The well-known *cog/cog* mice are famous for (and receive their name from) “*co*ngenital *g*oiter” [61]. The *cog-TG* gene encodes the mouse Tg-L2263P protein [66], which is misfolded and defective for intracellular transport from the ER to the Golgi complex [55,67]. Accumulation of the misfolded Tg induces ER stress that includes a marked elevation of ER chaperones and oxidoreductases [68,69] accompanied by massive ER expansion (Figure 2). A hypothyroid rat model known as *rdw/rdw* (encoding Tg-G2298R [47,70]) also exhibits dramatic ER stress response and massive ER expansion triggered by the misfolded rdwTg [71,72], resulting in an increase of cell size of individual thyrocytes [73,74]. However, the overall thyroid of *rdw/rdw* rats has been characterized as hypoplastic or atrophic [73,75], in contrast with the goiter of *cog/cog* mice (in which thyroid tissue ultimately weighs > 20-fold greater than normal [61]).

The reason for why hypothyroid *rdw/rdw* rats do not develop a goiter has been a research mystery for decades. A hypothesis was initially presented that the failure of thyroid gland growth may be secondary to thyrocyte cell death in *rdw/rdw* rats [72]. More specifically, the thinking was that thyroid enlargement in this animal model might be limited by an increased proteotoxicity of rdwTg as compared to goitrogenic *TG* variants, e.g., cogTg. However, it was later discovered that widespread thyrocyte cell death is a finding that is not exclusive to *rdw/rdw* rats, as this also occurs in *cog/cog* mice as well as a human patient with a similar biallelic *TG* mutation [76].

**Figure 3:**
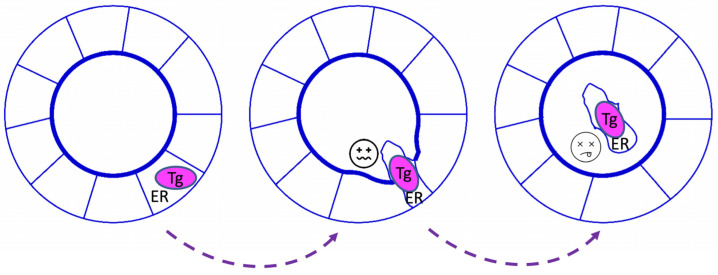
The mechanism of thyroid hormones synthesis in hypothyroidism with defective thyroglobulin. Thyrocytes under massive ER stress (*left*) lose epithelial cell-cell contact, likely from diminished cell junctional integrity, and progress to cell death (*right*). Dying thyrocytes that have been extruded into the follicle lumen eventually disintegrate and their macromolecular components are exposed to iodination and ingestion by surrounding live thyrocytes.

It is not easy to compare *cog/cog* thyroids in the AKR/J mouse strain background versus *rdw/rdw* thyroids in the Wistar-Imamichi rat strain background, for obvious reasons. Recently, an *rdw/rdw* knock-in model was generated in the C57BL6J mouse background using CRISPR/Cas9 technology [74]. Nowadays, *cog/cog* mice ordered from JAX laboratories are also distributed in the same strain background, making it possible to perform side-by-side examination of the cell biological and physiological impact of the two Tg mutants in the same genetic background [74]. Comparative studies of the two models have demonstrated that *rdw/rdw* (knock-in) mice do not exhibit greater ER stress or thyrocyte cell death compared to *cog/cog* mice, and both strains readily exhibit widespread thyrocyte cell death (Figure 3). Additionally, the proliferation of thyroid cells in *rdw/rdw* (knock-in) mice is not less than that found in *cog/cog* mice. As a result, the development of a goiter was not impaired in *rdw/rdw* (knock-in) mice when compared to *cog/cog* mice, indicating that the Tg-G2298R missense mutant is not intrinsically more proteotoxic than the Tg-L2263P mutant [74]. Rather, the absence of goiter in *rdw/rdw* rats appears to be linked primarily to strain-specific differences in thyroid gland growth [74]. Specifically, the *rdw/rdw* rats exhibit a failure to sustain thyroid cell proliferation despite ongoing TSH stimulation caused by primary hypothyroidism.

Simple goiter has been characterized as either a compensatory response to insufficient thyroid hormone production or as a maladaptation [77]. Interestingly, it has been found that patients bearing biallelic *TG* mutation with a large goiter could ultimately achieve nearly normal levels of thyroid hormone even when treatment with levothyroxine was not implemented (due to noncompliance or other reasons) [78,79]. Similarly, *cog/cog* mice were also found to be able to ‘spontaneously’ correct their serum T_4_ levels to nearly normal in the absence of levothyroxine treatment. The rise in serum T_4_ levels occurs slowly, and appears to parallel the growth of the thyroid gland [80]. Indeed, long-standing goiters resulting from untreated biallelic *TG* mutations have been reported to be associated with an increased risk of thyroid cancer [81,82,83]. It is conceivable that adenomatous and even malignant transformation might emerge from a prolonged hyperproliferative state, and this has been suggested based on studies in Wistar Hannover GALAS Rats (encoding Tgc.749−1G > T [84]), which display thyroid focal hyperplastic lesions in later life [85].

A comparative evaluation of the thyroids of untreated *cog/cog* mice, and a patient bearing homozygous Tg-W2346R, as well as *rdw/rdw* rats, revealed an unusual pathway of thyroid hormone synthesis that was shared by each case. Specifically, in all three species, thyrocyte cell death was observed, with entry of the dead, ER-stressed thyrocytes into the lumen of thyroid follicles still surrounded by other living thyrocytes [76]. This can lead to the iodination of proteins derived from decaying cells contained within the thyroid follicle lumen (which includes the mutant Tg protein), followed by the cannibalism of the iodinated luminal detritus of the dead thyrocytes (Figure 3). From the iodoproteins generated by iodination of the proteome of dead thyrocytes, some thyroxine can be formed within mutant Tg itself [76], although this is likely to be far less efficient than normal thyroid hormonogenesis. Interestingly, the compromised efficiency of iodoprotein synthesis could be partially improved with excess iodide supplement in a goat model [86] and in human patients [87].

Why are dead thyrocytes found within the thyroid follicle lumen? Interestingly, it has been observed that cell death in other epithelial tissues (including renal tubular epithelial cells, mammary epithelial cells, bronchial epithelial cells, etc.) results in dead cell extrusion selectively to the apical side of the epithelium [88,89,90]. Thyroid follicles are similar, organized as a classically polarized epithelial monolayer, with typical tight junctions and gap junctions [91], such as adherens junctions [92] and desmosomes [93]. Adherens junctions primarily consist of protein complexes between the transmembrane protein cadherin and intracellular catenin, and these complexes are linked to the actin cytoskeleton [94]. The formation of adherens junctions is a prerequisite for the proper assembly of tight junctions [95]; indeed, cadherin and other cell-adhesion proteins mediate many facets of epithelial morphogenesis [96]. Thyroid cells express E-cadherin and thyroid (and kidney)-specific cadherin-16 [97], which also contributes to the apical–basal follicular polarization [98] that is essential to efficient thyroid hormonogenesis [21,22]. On the other hand, decreased expression of cadherins favors epithelial cell disaggregation, as happens in epithelial-mesenchymal transition that can occur during the development of various cancers [99]. It is thought that thyroidal ER stress (triggered pharmacologically, and potentially also pathologically from Tg misfolding) induces a thyroid de-differentiation response [52,100] that includes not only gene products directly implicated in thyroid hormonogenesis, but also diminished expression of E-cadherin and cadherin-16. This may bring about thyrocyte cell shape change, as well loss of cortical actin, formation of stress fibers, and significantly, a loss of epithelial cell-cell contact. Such changes in the thyrocyte cytoskeleton and accompanying loss of cell-cell junctional contacts might account for why—in patients and animal models with biallelic *TG* mutation—dead ER-stressed thyrocytes are observed to be extruded apically into the thyroid follicular lumen [76]. Indeed, not only may dead thyrocytes be released into the thyroid follicular lumen, but it is also conceivable that live follicular epithelial cells might also enter the thyroid follicle lumen, although it remains to be tested the extent to which this occurs, and if so, whether the driving mechanisms are the same.

Interestingly, in the early days of life, *rdw/rdw* rats actually show mild thyroid gland enlargement compared to that of wildtype animals, and this corresponds to a short period in the life of the mutant animals during which serum T_4_ levels are actually increasing (although still abnormally low). This stage of life is then followed by a sustained period during which a decrease of both thyroid gland size and serum T_4_ levels is observed [76]. Although the production of thyroid hormones via the iodination of disintegrating thyrocytes in the follicular lumen is very inefficient, nevertheless this highly unusual mechanism is a key source for providing thyroid hormone synthesis, and blocking this source is actually lethal to the animals [76]. Thus, in comparing *cog/cog* mice to *rdw/rdw* rats, it appears that the goiter is necessary in order to provide a continuous supply of thyrocytes, that upon entry and disintegrations within the follicle lumen, ultimately provide the substrate that allows for ongoing thyroid hormone biosynthesis. Conversely, failure to grow the thyroid gland causes *rdw/rdw* rats, and probably some human patients, to become depleted of the substrate needed to sustain thyroid hormonogenesis.
